# Geographic access to federally qualified health centers before and after the affordable care act

**DOI:** 10.1186/s12913-022-07685-0

**Published:** 2022-03-23

**Authors:** Caroline L Behr, Peter Hull, John Hsu, Joseph P Newhouse, Vicki Fung

**Affiliations:** 1 Harvard Medical School, Boston, USA; 2 Massachusetts General Hospital, Boston, USA; 3 Brown University, Providence, USA; 4 National Bureau of Economic Research, Cambridge, USA; 5 T. H. Chan School of Public Health, Boston, USA; 6 Harvard Kennedy School, Cambridge, USA

**Keywords:** Access to care, Underserved populations, Primary care safety net, Rural health, Health disparities

## Abstract

**Background:**

The Affordable Care Act (ACA) increased funding for Federally Qualified Health Centers (FQHCs). We defined FQHC service areas based on patient use and examined the characteristics of areas that gained FQHC access post-ACA.

**Methods:**

We defined FQHC service areas using total patient counts by ZIP code from the Uniform Data System (UDS) and compared this approach with existing methods. We then compared the characteristics of ZIP codes included in Medically Underserved Areas/Populations (MUA/Ps) that gained access vs. MUA/P ZIP codes that did not gain access to FQHCs between 2011–15.

**Results:**

FQHC service areas based on UDS data vs. Primary Care Service Areas or counties included a higher percentage of each FQHC’s patients (86% vs. 49% and 71%) and ZIP codes with greater use of FQHCs among low-income residents (29% vs. 22% and 22%), on average. MUA/Ps that gained FQHC access 2011–2015 included more poor, uninsured, publicly insured, and foreign-born residents than underserved areas that did not gain access, but were less likely to be rural (*p* < .05).

**Conclusions:**

Measures of actual patient use provide a promising method of assessing FQHC service areas and access. Post-ACA funding, the FQHC program expanded access into areas that were more likely to have higher rates of poverty and uninsurance, which could help address disparities in access to care. Rural areas were less likely to gain access to FQHCs, underscoring the persistent challenges of providing care in these areas.

**Supplementary Information:**

The online version contains supplementary material available at 10.1186/s12913-022-07685-0.

## Background

Federally Qualified Health Centers (FQHCs) are a critical component of the US primary care safety net [1]. The Affordable Care Act established the Community Health Center Fund (CHCF) to support the expansion of FQHCs between 2011–2015, allocating $11 billion over five years. The creation of the CHCF, along with the ACA’s Medicaid expansion, led to a dramatic increase in both the funding and growth of FQHCs [2]. FQHC delivery sites are owned by FQHC Grantee organizations (Grantees), which may operate multiple FQHC sites. During the initial CHCF authorization period from 2010 to 2015, the number of Grantees grew from 1,124 to 1,375, the number of FQHC delivery sites increased from 6,949 to 9,754, and the total number of patients served grew 25%, from 19.5 million to 24.3 million [3]. Despite the new funding and growth in sites and patients, there remains no standard by which to assess this growth – in part because of uncertainty on how to best define the area served by an FQHC. In this paper we seek to assess how the post-ACA expansion of FQHC sites expanded geographic access to care and for which populations using data on the locations of FQHC patients.

Expansion of FQHC sites to underserved areas could help reduce geographic health disparities. FQHCs are required to include Medically Underserved Areas (MUAs) or Populations (MUPs), which are designated by the Health Resources and Services Administration (HRSA) or specially designated on the state level. They are also required to provide care to patients regardless of ability to pay, making them a critical source of care for the uninsured. Geographic proximity to FQHCs has been associated with a greater probability of having a usual source of care and having physician visits in the past year [4–6]. Moreover, some studies have demonstrated that low-income patients receiving care from FQHC- vs. non-FQHC providers have better care quality, such as receipt of cancer screening, better access to dental care, better diabetes outcomes, and fewer preventable hospitalizations and emergency room visits [6–14].

The post-ACA expansion of the FQHC program has been shown to improve primary care access in shortage areas; provider availability has improved and more patients have a usual source of care [13, 15]. Findings are limited and mixed, however, on which types of underserved areas were more likely to benefit from recent expansions of the FQHC program [16, 17]. A contributing factor to these varied findings is the challenge of defining an FQHC’s service area.

Data on FQHCs are collected on the Grantee level, but organizations might have up to hundreds of FQHC delivery sites. Depending on the number of FQHCs and the range of locations operated by a Grantee, these service areas vary considerably in size and can encompass non-contiguous geographic areas. In addition, patients’ use of FQHCs could vary as a function of both FQHC capacity and the local availability of non-FQHC providers. Thus, access is likely predicated on patient need and behavior given available options and might not map cleanly to administrative geographic boundaries.

Previous studies have used pre-existing geographic boundaries, such as counties [18] or Primary Care Services Areas (PCSAs) as defined by the Dartmouth Atlas [17], while others have sought to define service areas based on commuting time from FQHC delivery sites [16]. These approaches could be poor reflections of actual access, as reflected in patient use. They may underestimate the care provided by centers, and hinder evaluations that seek to characterize the populations gaining access to FQHCs.

In this analysis we evaluated the performance of an approach adapted from hospital market share definitions to empirically define the service areas of FQHCs using information on the ZIP code locations of patients receiving care at FQHCs. We then applied this approach to examine the characteristics of medically underserved areas that gained access to an FQHC during the initial authorization of the CHCF.

## Methods

### Data sources and study population

The data for this study are from 2010–2015 Uniform Data System (UDS) for FQHCs. The UDS requires FQHCs to report information at the FQHC Grantee level, including yearly counts of each FQHC Grantee’s patients by residential five-digit ZIP code, Grantee characteristics, and addresses of FQHC delivery sites by year. To limit this analysis to FQHCs accessible to the general population, we focus on the 1,158 Grantees that received Community Health Center Funding located in the 50 United States and Washington, D.C., excluding 109 special population health centers designated as migrant health centers, healthcare for the homeless, or public housing primary care, between 2010–2015. We also excluded 22 Grantees with missing ZIP code data for more than 50% of their patients.

For area-level characteristics, we obtained data from the American Community Survey, the Area Health Resource File, HRSA data on geographic areas designated as Medically Underserved Areas/Populations (MUA/P) or Health Professional Shortage Areas (HPSA), and US Department of Agriculture Rural–Urban Commuting Area (RUCA) Codes.

### FQHC service area definitions

We adapted a previously used hospital market share definition method to define FQHC Grantee service areas (“FQHC service areas”) based on UDS patient count data, with the goal of identifying ZIP codes with a high degree of FQHC usage [19]. This method allows for the definition of service areas based on actual patient use while accounting for the complexity of the Grantee-level (multi-site) data that is available for FQHCs. To implement this approach, we included any ZIP code that contributed at least 3% of each FQHC Grantee’s patient population, plus additional ZIP codes (from largest to smallest by patient count) until at least 40% of the Grantee’s patients were included (base approach). In addition, we modified this base approach by also including in each Grantee’s service area the ZIP codes of all the organization’s individual FQHC delivery site locations and all ZIP codes where at least 40% of the ZIP code’s population was served by the Grantee (modified approach). This 40% threshold was used to account for rural ZIP codes that could have small population counts but high local use of FQHCs. In sensitivity analyses, we compared the base approach and the modified approach and examined other thresholds for including ZIP codes with a high proportion of residents served (30% and 50%); findings were similar.

To compare the performance of the UDS-based service areas with two other common approaches to define service areas, we also defined FQHC service areas using the county or Primary Care Service Areas (PCSAs, 2013) that included the ZIP code with the largest proportion of a Grantee’s patients. We linked ZIP codes with counties using the 2015 Department of Housing and Urban Development and United States Postal Service ZIP Code Crosswalk files [20]. PCSAs were developed by the Dartmouth group using data on Medicare ambulatory care visits, validated for younger populations using Medicaid data, and then adjusted to compromise continuous ZIP codes [21]. There were 6,871 PCSAs in 2013.

We compared the proportion of each FQHC Grantee’s patients in 2015 who were included in the service areas defined by each of the three approaches as well as the average number of ZIP codes included in each definition. We then examined FQHC use rates per population for the included ZIP codes in each definition to assess whether there were differences across service area definitions in the area-level FQHC penetration. Because 92% of all FQHC patients have incomes under 200% FPL [3], we calculated the use rate as the number of patients from each ZIP code as reported in the UDS, divided by the Zip Code Tabulation Area (ZCTA) population under 200% FPL, which we obtained from the American Community Survey.

To visually examine differences across service area definitions, we mapped ZIP codes that were included in FQHC service areas in 2010 and newly included ZIP codes between 2011–2015 using QGIS version 3 and 2010 Census TIGER/Line Shapefiles for ZCTAs [22]. To assess how these areas corresponded with the location of FQHC delivery sites, we geocoded delivery site addresses using OpenStreetMap [23] and MMQGIS package using data from the UDS. We illustrate our methods for Louisiana.

### Changes in FQHC service areas

We identified MUA/P areas that were classified as FQHC services areas in 2010, those newly classified as FQHC service areas between 2011–2015, and those not included in FQHC service areas in any year 2010–2015. This category of newly classified service area ZIP codes allows us to identify the geographic impact of both new delivery sites and the expansion of existing delivery sites in capacity or geographic reach post-ACA funding. This analysis was limited to ZIP codes that were designated as MUA/Ps by HRSA because FQHCs are required to serve a MUA or MUP. MUAs are defined as geographic areas that have a shortage of primary care services, and MUPs are specific populations within an established geographic area that have a shortage of primary care services (e.g., low-income populations, populations experiencing homelessness) [24]. MUA/Ps are designated by HRSA (and occasionally by states) at the Census tract level, county level, and minor civil divisions. To link these with ZIP code level data in the UDS, we used crosswalk files available from the U.S. Department of Housing and Urban Development (HUD) [20]. All ZIP codes that contained an MUA/P in 2015 or were contained in an MUA/P were included in our analyses.

### Analysis

We examined the characteristics of MUA/P ZIP codes that were included in FQHC service areas in 2010 (pre-ACA) vs. 2011–2015 (post-ACA) vs. MUA/P ZIP codes not included anytime 2010–2015. To characterize the sociodemographic characteristics of MUA/P ZIP codes we used five-year estimates from the 2011–2015 American Community Survey to examine sociodemographic characteristics, including age, sex, race, ethnicity, insurance coverage mix (percentage with public insurance, private insurance, or uninsured), urbanicity, and region. To assess urbanicity, we used the four category RUCA classification as of August 2020 to designate ZIP codes as urban, large rural, small rural, and isolated [25].These designations are based on population density and commuting patterns. We used the 2015 Area Health Resource File to identify ZIP codes with Rural Health Clinics. We also used 2015 HRSA data on Health Professional Shortage Areas (HPSAs), to identify ZIP codes in primary care HSPAs, mental health HPSAs, and dental HSPAs. In this definition we used all designated HPSAs, including minor civil divisions, census tracts, counties, and single-point HPSAs, and used 2015 HUD crosswalk files to link these areas with ZIP codes. Medicaid-expansion ZIP codes were defined including ZIP codes in all states that expanded Medicaid prior to 2016 [26].

To compare the characteristics of MUA/P ZIP codes that were newly included in FQHC service areas 2011–2015 vs. not included anytime between 2010–2015, we used multivariate logistic regression.

## Results

**Fig. 1:**
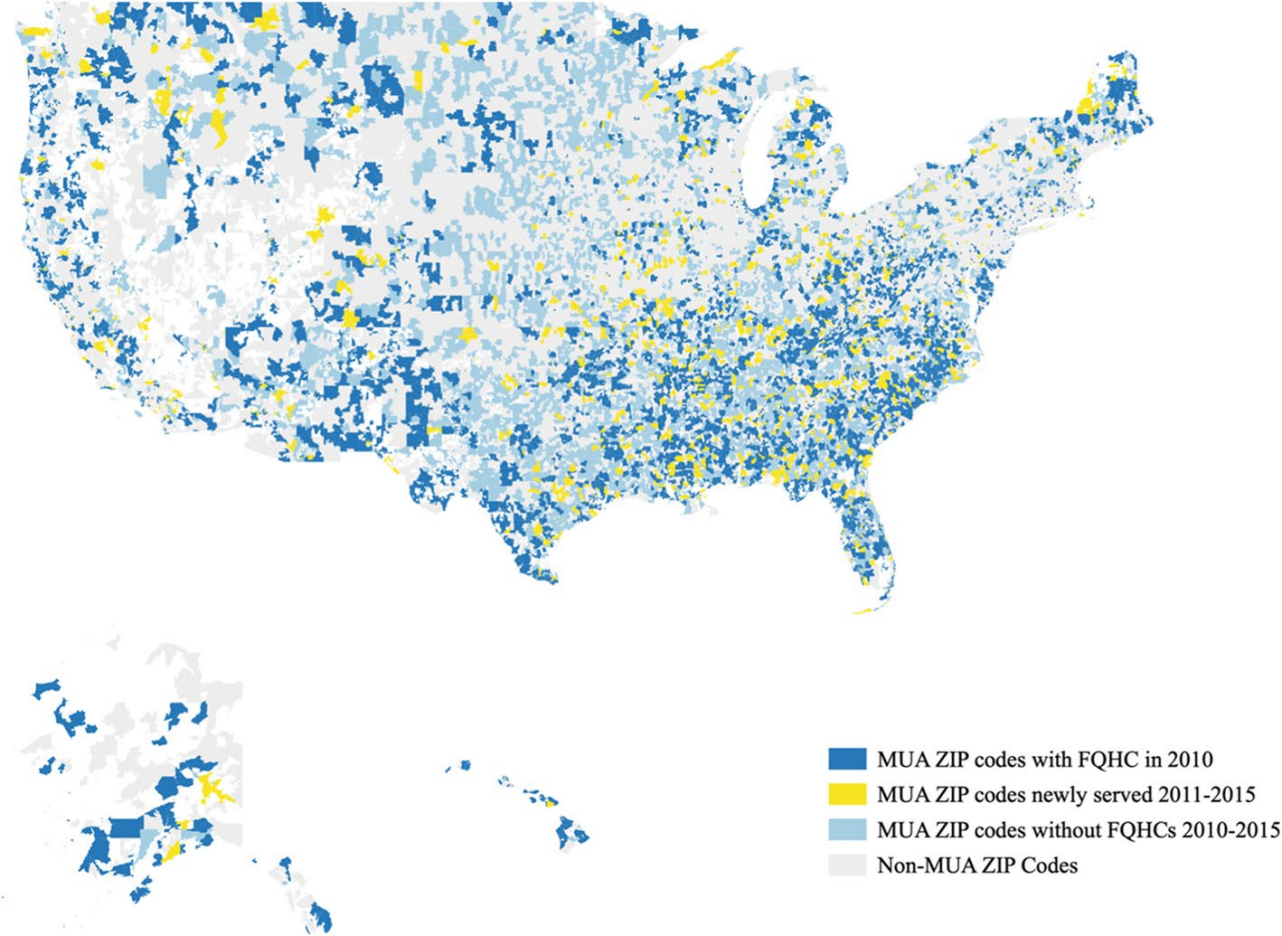
ZIP codes with MUA/P designations included in FQHC Service Areas in 2010 vs. 2011–2015 using UDS-based definition

There were 11,369 ZIP codes that were designated as MUA/Ps in 2015, and thus potentially eligible to receive an FQHC. Using the UDS-based definition, 4,600 of these ZIP codes were in an FQHC service area in 2010 (representing 64% of the MUA/P population), another 1,242 ZIP codes (13% of the MUA/P population) were newly included in an FQHC service area between 2011–2015, and 5,527 ZIP codes (23% of the MUA/P population) were not included in an FQHC service area before 2011 nor by 2015. Figure 1 shows a map of these ZIP codes for the United States.

### Comparison of service area definitions

The UDS-based approach captured a larger percentage of patients who received care in an FQHC (86% [IQR: 80–94%]), compared with both the PCSA (49% [IQR: 30–68%]) and county-based definitions (71% [IQR: 54–92%], Table 1), while on average including a similar or fewer number of ZIP codes (8.9 [IQR: 6–11] vs. 8.0 [IQR: 3–11] for PCSA and 24.3 [IQR: 8–31] for county). Furthermore, among the ZIP codes included in empirically defined FQHC service areas, there was a higher rate of FQHC use among the residents with incomes < 200% FPL compared with the other two approaches (roughly 29%, vs. 22% and 22%).

**Table 1:** Comparison of UDS-based approach for defining FQHC service areas vs. Primary Care Service Area and County (2015)

	UDS-based service areas	Primary Care Service Areas^b^	County^c^
Mean [IQR] # ZIP codes per FQHC included in service area	8.9 [6-11]	8.0 [3-11]	24.3 [8-31]
Mean [IQR] proportion of FQHC patients included	86% [80–94]	49% [30–68]	71% [54–92]
Total # of ZIP codes included in FQHC service areas	8,713	8,568	10,105
Total population included in FQHC service areas (millions)	167.1	107.7	215.7
% of FQHC service area population with income < 200% FPL that visited an FQHC^a^	29%	22%	22%
% of delivery site ZIP codes included in FQHC service areas	100%	53%	74%

^a^Estimated by calculating total number of patients from FQHC’s defined service area divided by population of service area under 200% FPL

^b^Defined by identifying ZIP codes with largest proportion of patients per FQHC, linking ZIP code to associated PCSA, and then including all ZIP codes linked that PCSA in the service area

^c^Defined by identifying ZIP codes with largest proportion of patients per FQHC, linking ZIP code to the associated county, and then including all ZIP codes linked that PCSA in the service area

**Fig. 2:**
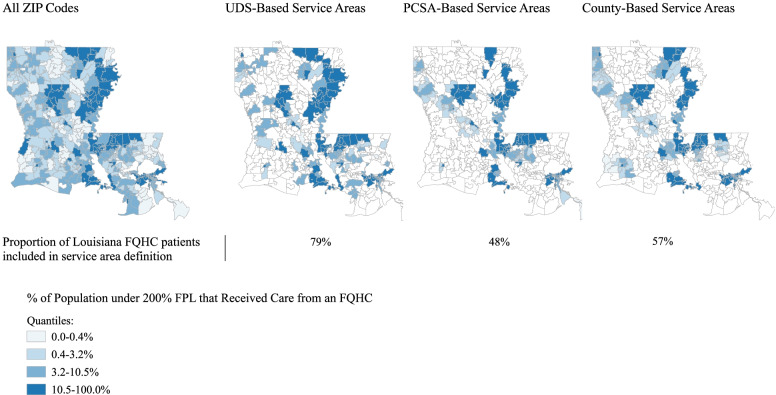
Density of FQHC Patients in Louisiana, 2015: All ZIP Codes vs. Three Service Area Definitions

Figure 2 compares these three approaches in Louisiana, a state with one of the largest increases in the total number of FQHC grantees. The UDS-based service areas included 79% of FQHC patients in Louisiana in 2015, compared with 48% and 57% of patients in the PCSA and county definitions, respectively (Supplemental Table 1), as well as a greater proportion of ZIP codes in the highest quartile of FQHC use (roughly 74%, vs. 65% and 72%; see Fig. 2).

### Characteristics of areas served in 2010

Among 4,600 ZIP codes included in FQHC service areas in 2010 using the UDS-based definition, the mean population was 20,577 (Table 2). Within these ZIP codes, 63.5% of the population was White, 20.4% Black, 4.3% Asian, and 16.3% of residents were born outside the United States. We found that 44.5% of patients living in these ZIP codes had incomes below the 200% FPL, with 16.6% of residents uninsured, and 37.6% on public insurance. The majority of these 2010 ZIP codes were urban (50.8%), and regionally the highest proportion were in the South (46.1%).

**Table 2:** Characteristics of MUA/P ZIP Codes included and not included in FQHC service areas using UDS-based approach

Characteristics	ZIP Codes with FQHCs in 2010	Newly Included in Service Areas ZIP Codes 2011–2015	ZIP Codes without FQHCs 2010- 2015	aOR (Newly Included vs. Not Included in Service Areas)
Total ZIP codes	4,600	1,242	5,527	-
Mean ZIP population (SD)	20,577 (18,598)	14,820 (14,508)	6,471 (8,918)	-
Total population (2015, millions)	94.7	18.4	35.8	
*Population-Level Characteristics:*				
Age: < = 20 years old	26.8%	25.8%	25.2%	0.99 [0.98–1.00]
20–64 years old	60.1%	59.6%	58.5%	**-**
65 + years old	13.1%	14.6%	16.3%	**0.98 [0.97–0.99]**
Race: White	63.5%	75.1%	81.5%	-
Black	20.4%	13.6%	9.5%	**1.09 [1.04–1.14]**
Asian	4.3%	3.7%	2.5%	0.87 [0.69–1.1]
American Indian/ Alaska Native	0.9%	0.7%	1.3%	**0.69 [0.58–0.81]**
Native Hawaiian/ Pacific Islander	0.2%	0.1%	0.1%	0.75 [0.21–2.7]
Other	7.6%	4.0%	2.5%	1.00 [0.81–1.23]
Two Plus	3.1%	2.7%	2.5%	**1.47 [1.11–1.94]**
Ethnicity: Hispanic	25.7%	15.7%	10.9%	**0.87 [0.80–0.95]**
Place of birth: Foreign born	16.3%	10.7%	7.3%	**1.52 [1.28–1.81]**
HH income: < 200% FPL	44.5%	37.9%	33.3%	**1.32 [1.10–1.57]**
Insurance type: Private	53.7%	61.6%	65.5%	**-**
Public	37.6%	33.1%	32.6%	**1.14 [1.04–1.26]**
Uninsured	16.6%	14.4%	12.5%	**1.19 [1.05–1.34]**
*ZIP Code-Level Characteristics:*				
Urbanicity: Urban	50.8%	33.2%	20.6%	-
Large rural	23.2%	32.0%	36.4%	**0.53 [0.44–0.64]**
Small rural	9.1%	13.1%	13.1%	**0.56 [0.44–0.72]**
Isolated rural	16.9%	21.7%	29.8%	**0.45 [0.36–0.56]**
Rural Health Clinic	42.7%	49.6%	58.2%	**0.72 [0.62–0.83]**
HSPA: Primary Care	74.6%	69.3%	65.1%	1.08 [0.92–1.27]
Mental Health	72.0%	72.1%	67.6%	**1.38 [1.15–1.65]**
Dental Health	71.5%	66.3%	58.5%	**1.37 [1.16–1.61]**
Medicaid Expansion State	57.9%	51.0%	51.9%	1.06 [0.89–1.26]
Northeast	14.8%	9.8%	11.2%	-
South	46.1%	51.5%	45.9%	1.02 [0.78–1.34]
Midwest	18.4%	22.6%	30.7%	1.12 [0.87–1.46]
West	20.7%	16.0%	12.1%	**1.57 [1.17–2.10]**

Analysis performed in STATA v.14.0. Adjusted odds ratios for continuous variables were calculated for a 10-percentage point difference. All characteristics are population-weighted means

### Comparing newly served and unserved areas

ZIP codes that were newly included vs. not included in FQHC service areas were more likely to have a larger proportion of Black residents (13.6% vs. 9.5%; OR 1.09 [95% CI: 1.04–1.14] for a 1 percentage point increase), foreign-born residents (10.7% vs. 7.3%, OR 1.52 [1.28–1.81]), residents with incomes below 200% FPL (37.9% vs. 33.3%, OR 1.32 [1.10–1.57]), uninsured (14.4% vs. 12.5%, OR 1.19 [1.05–1.34]), and publicly insured (33.1% vs. 32.6%, OR 1.14 [1.04–1.26])) residents (Table 2). ZIP codes that were newly included vs. not included in FQHC service areas were also more likely to be shortage areas, with 72.1% vs. 67.6% in mental health HPSAs (OR 1.38 [1.15–1.65]), and 66.3% vs. 58.5% in dental health HPSAs (OR 1.37 [1.16–1.61]).

MUA/P ZIP codes that were newly included in FQHC service areas were less likely to be in rural areas (e.g., 21.7% vs. 29.8% in isolated rural areas, OR 0.45 [0.36–0.56]. These areas also had a lower proportion of residents that were age 65 + (14.6% vs 16.3, OR 0.98 [0.97–0.99]) or American Indian/Alaska Native (0.7% vs. 1.3%, OR 0.69 [0.58–0.81]) patients vs. areas not included in FQHC service areas.

## Discussion

In this study, we assessed how the ACA changed geographic access to FQHC services by adapting a method used to assess hospital market share to the definition of FQHC service areas based on public patient use data reported by FQHCs to HRSA. This approach captures a greater proportion of FQHC patients and identifies ZIP codes with greater FQHC use, on average, than previously used methods that use pre-existing geographic boundaries or require contiguous geography. Using this approach, we found that the initial expansion of the FQHC program under ACA funding increased access to safety net primary care services for about 18.4 million residents living in medically underserved ZIP codes in 2015.

In contrast to other studies that have examined changes in geographic access to FQHCs, our approach did not measure changes in FQHC service areas solely associated with the location of new delivery sites, which could vary in capacity and service offerings [16, 17]. While providing funds for new delivery sites was a crucial component of CHCF funding, FQHCs could also use the funding to expand service offerings, improve infrastructure, and hire additional staff [2], enabling existing delivery sites to expand capacity and geographic reach. Our measure of FQHC service areas and their change over time could thus better reflect changes in access to and use of FQHCs associated with ACA-related revenue increases than prior measures, as well as changes in local need for care or local supply of non-FQHC care.

Our study adds to the limited evidence on changes in area-level access to FQHCs following the ACA. Similar to one national study, which compared FQHC service areas in 2014 to 2007 based on PCSAs, we found that areas newly included in service areas had a lower proportion of residents below federal poverty standards than pre-ACA FQHC service areas [17]. However, among medically underserved areas that did not previously have access to an FQHC, we found newly served ZIP codes were more likely to have higher rates of poverty, uninsurance, a greater proportion of Black residents, and were more likely to be designated as mental health and dental health professional shortage areas. These findings suggest that the growth of the FQHC program post-ACA expanded access to medically underserved areas with populations that could face greater structural barriers to health care access (e.g., related to discrimination, socioeconomic status, provider supply), and to groups that were disproportionately impacted by the COVID-19 pandemic. FQHCs have faced many financial challenges during the pandemic that led to some temporary closures of delivery sites and staff downsizing, but FQHCs have also served as critical providers of COVID-19 testing, care, and vaccinations for racial/ethnic minority and low-income patients [27, 28].

Underserved rural vs. urban areas, however, were less likely to be included in FQHC service areas post-ACA, which may reflect compounding challenges of staffing, reimbursement, and volume that health care facilities face in rural settings. Rural residents receiving care at FQHCs have previously been found to be more likely to receive certain preventive services, such as Pap smear and pneumococcal vaccinations compared with the general rural population [29]. Greater expansion of FQHCs or similar interventions in rural areas could help address health disparities present in rural communities and potentially address reductions in the availability of emergency and hospital care [30]. These findings support future investigations into barriers in the development of rural FQHCs and the consideration of policy interventions to better target these rural communities.

The Community Health Center Fund has been reauthorized by Congress multiple times at levels of funding similar to that of the ACA [31]. It comprises a growing share of federal Sect. 330 funds for FQHCs, and is currently authorized through September 30, 2023 [32]. In addition, the American Rescue Plan included $7.6 billion in emergency COVID-19 funding for FQHCs. This continued funding has contributed to sustained growth of the FQHC program, which served 29.8 million patients in 2019 [33].

### Limitations

This study has several limitations. We defined service areas based on aggregate data at the FQHC level; using these data, we are not able to quantify the regularity or types of services received by individual patients, which could improve measures of service areas and access to care. We also were not able to directly capture a distinction between newly offered services or new delivery sites and services that shifted to serve new geographic areas. In addition, we could not identify patients with visits to multiple FQHCs and we could overestimate use of FQHCs within a given ZIP code; in prior work using claims data, however, we found that use of multiple FQHCs was uncommon [9].

## Conclusions

Through data on patient use, the findings of this study suggest that the ACA increased access to safety net primary care to underserved populations that experience significant health disparities – but gaps for rural areas remain.

## Supplementary Information

**Additional file 1.**

## Data Availability

FQHC UDS data are available from the corresponding author on reasonable request. American Community Survey data are available through the U.S. Census Bureau, https://www.census.gov/programs-surveys/acs/data.html. Area Health Resource Files and Health Professional Shortage Areas available through the Health Resources and Services Administration Data Warehouse; https://data.hrsa.gov/data/download, Rural–Urban Commuting Codes are available from the U.S. Department of Agriculture Economic Research Service, https://www.ers.usda.gov/data-products/rural-urban-commuting-area-codes/.

## Abbreviations

ACA: Affordable Care Act
CHCF: Community Health Center Fund
FQHC: Federally Qualified Health Center
HPSA: Health Professional Shortage Area
HUD: U.S. Department of Housing and Urban Development
MUA: Medically Underserved Area
MUP: Medically Underserved Population
PCSA: Primary Care Service Area
RUCA: Rural–Urban Commuting Area
UDS: Uniform Data System
ZCTA: Zip Code Tabulation Area
