# Supplementary material for: Geographic access to federally qualified health centers before and after the affordable care act
Source: BMC Health Serv Res. 2022 Mar 23;22:385. doi: 10.1186/s12913-022-07685-0 (PMC8942056; doi:10.1186/s12913-022-07685-0)
Supplement: Supplementary file 1 — Additional file 1. [file 12913_2022_7685_MOESM1_ESM.docx]

**Supplemental File:** Geographic Access to Federally Qualified Health Centers Before and After the Affordable Care Act

**Supplemental Table 1: Louisiana Comparison of UDS-based approach for defining FQHC service areas vs. Primary Care Service Area and County (2015)**

|  | UDS-based service areas | Primary Care Service Areas^2^ | County^3^ |
| --- | --- | --- | --- |
| % of Highest Quartile ZIP codes included | 74% | 65% | 72% |
| Proportion of Louisiana FQHC patients included | 79% | 48% | 57% |
| Total # of ZIP codes included in FQHC service areas | 179 | 123 | 268 |
| Total population included in FQHC service areas (millions) | 3.0 | 2.1 | 2.6 |
| % of FQHC service area population with income <200% FPL that visited an FQHC^1^ | 19% | 19% | 18% |
| % of delivery site ZIP codes included in FQHC service areas | 100% | 52% | 56% |

1 Estimated by calculating total number of patients from FQHC’s defined service area divided by population of service area under 200% FPL

2 Defined by identifying ZIP codes with largest proportion of patients per FQHC, linking ZIP code to associated PCSA, and then including all ZIP codes linked that PCSA in the service area.

3 Defined by identifying ZIP codes with largest proportion of patients per FQHC, linking ZIP code to the associated county, and then including all ZIP codes linked that PCSA in the service area.

**Supplemental Table 2: Characteristics of MUA/P ZIP Codes Previously Included in Service Areas vs. Newly Included in Service Areas using UDS-based approach**

| Characteristics | aOR (Newly included 2011-2015 vs. Included in Service Areas in 2010) |  |  |
| --- | --- | --- | --- |
| *Population-Level Characteristics:* |  |  |  |
| Age: <= 20 years old | 1.01 [1.0-1.03] |  |  |
| 20-64 years old | 1.01 [1.0-1.03] |  |  |
| 65+ years old |  |  |  |
| Race: White | - |  |  |
| Black | **0.93 [0.89-0.97]** |  |  |
| Asian | 0.96 [0.79-1.16] |  |  |
| American Indian/ Alaska Native | **0.68 [0.55-0.85]** |  |  |
| Native Hawaiian/ Pacific Islander | **0.18 [0.04-0.78]** |  |  |
| Other | 1.0 [0.84-1.2] |  |  |
| Two Plus | 0.98 [0.71-1.36] |  |  |
| Ethnicity: Hispanic | **0.86 [0.79-0.93]** |  |  |
| Place of birth: Foreign born | 1.13 [0.98-1.31] |  |  |
| HH income: <200% FPL | **1.27 [1.07-1.52]** |  |  |
| Insurance type: Private | **-** |  |  |
| Public | 0.97 [0.84-1.13] |  |  |
| Uninsured | **0.65 [0.59-0.72]** |  |  |
| *ZIP Code-Level Characteristics:* |  |  | |
| Urbanicity: Urban | - |  |  |
| Large rural | **1.8 [1.49-2.18]** |  |  |
| Small rural | **1.9 [1.49-2.42]** |  |  |
| Isolated rural | **1.87 [1.49-2.35]** |  |  |
| Rural Health Clinic | 1.13 [0.98-1.31] |  |  |
| HSPA: Primary Care | 0.84 [0.7-1.0] |  |  |
| Mental Health | 0.96 [0.8-1.15] |  |  |
| Dental Health | **0.77 [0.64-0.92]** |  |  |
| Medicaid Expansion State | 0.88 [0.74-1.04] |  |  |
| Northeast | **-** |  |  |
| South | **1.93 [1.51-2.47]** |  |  |
| Midwest | **1.76 [1.36-2.28]** |  |  |
| West | **1.69 [1.26-2.27]** |  |  |
| Analysis performed in STATA v.14.0. Adjusted odds ratios for continuous variables were calculated for a 10-percentage point difference. All characteristics are population-weighted means. | | |  |
